# Distinct Steps of Neural Induction Revealed by Asterix, Obelix and TrkC, Genes Induced by Different Signals from the Organizer

**DOI:** 10.1371/journal.pone.0019157

**Published:** 2011-04-29

**Authors:** Sonia Pinho, Pamela R. Simonsson, Katherine E. Trevers, Matthew J. Stower, William T. Sherlock, Mohsin Khan, Andrea Streit, Guojun Sheng, Claudio D. Stern

**Affiliations:** 1 Department of Cell and Developmental Biology, University College London, London, United Kingdom; 2 Department of Craniofacial Development, King's College London, London, United Kingdom; Laboratoire Arago, France

## Abstract

The amniote organizer (Hensen's node) can induce a complete nervous system when grafted into a peripheral region of a host embryo. Although BMP inhibition has been implicated in neural induction, non-neural cells cannot respond to BMP antagonists unless previously exposed to a node graft for at least 5 hours before BMP inhibitors. To define signals and responses during the first 5 hours of node signals, a differential screen was conducted. Here we describe three early response genes: two of them, Asterix and Obelix, encode previously undescribed proteins of unknown function but Obelix appears to be a nuclear RNA-binding protein. The third is TrkC, a neurotrophin receptor. All three genes are induced by a node graft within 4–5 hours but they differ in the extent to which they are inducible by FGF: FGF is both necessary and sufficient to induce *Asterix*, sufficient but not necessary to induce *Obelix* and neither sufficient nor necessary for induction of *TrkC*. These genes are also not induced by retinoic acid, Noggin, Chordin, Dkk1, Cerberus, HGF/SF, Somatostatin or ionomycin-mediated Calcium entry. Comparison of the expression and regulation of these genes with other early neural markers reveals three distinct “epochs”, or temporal waves, of gene expression accompanying neural induction by a grafted organizer, which are mirrored by specific stages of normal neural plate development. The results are consistent with neural induction being a cascade of responses elicited by different signals, culminating in the formation of a patterned nervous system.

## Introduction

In 1924, Spemann and Mangold performed one of the most important experiments in the history of embryology: they showed that transplantation of the dorsal lip of the blastopore of an amphibian embryo into the ventral side of a host embryo of a differently-pigmented species can induce a well organized ectopic axis derived mainly from cells of the host [1]. This experiment defined embryonic induction unambiguously as an influence from one group of cells that changes the fate of another [2]. It also introduced the concept of “organizer”, a group of cells that emits signals capable of both embryonic induction and patterning. Soon thereafter, a region with equivalent properties was discovered in birds and mammals to reside in Hensen's node, at the tip of the primitive streak [3]–[8] and in the embryonic shield of teleosts [9]–[11].

The activity of the organizer influences cells in both mesoderm and ectoderm. Exposure of mesoderm to organizer signals at the gastrula stage or later can “dorsalize” its fate so that prospective lateral plate cells give rise to somites [12]–[17]. A graft of the organizer adjacent to non-neural ectoderm can convert host prospective epidermis into neural plate, a process known as “neural induction” (for review see [18]). Both processes involve inhibition of BMP signaling in host cells by factors secreted by the organizer [15], [16], [19]–[25]. In the mesoderm, BMP inhibition is sufficient to dorsalize mesoderm cells even at late stages [15], [16]. In contrast, in the ectoderm BMP only acts alone within the future neural plate territory or its border – in regions not contiguous with the neural plate, additional signals are required for cells to respond to BMP or its antagonists [26]–[32].

In the chick embryo, a graft of Hensen's node can neuralize the extraembryonic epiblast of the area opaca until the end of gastrulation [33], [34]. BMP inhibitors alone cannot induce expression of any neural markers under the same conditions [26], [28]. Time-course experiments have suggested that other signals from Hensen's node are required for these cells to become sensitive to BMP inhibition. A graft of Hensen's node induces the pre-neural plate marker *Sox3* within 3 hours, but if the graft is removed before 12 hours, the induced expression is lost and no neural plate develops. In contrast, expression of *Sox3* can be maintained if a source of BMP inhibitors is presented following removal of the node at 5 hours, but not earlier [28]. These findings suggest that organizer-derived signals other than BMP inhibitors are required for epiblast cells to become responsive to BMP antagonism.

To identify these signals, we performed a differential screen between area opaca epiblast cells that had been exposed to a graft of Hensen's node for 5 hours and control cells from the contralateral side of the same embryo. The expression of about 10 genes was found to differ between the two conditions. Of these, we have previously described some novel genes like *ERNI* [35], *Churchill* [36] and *Calfacilitin* (Papanayotou et al., in preparation) and some genes that had previously been studied in other contexts: *Sox3* [35], *Dad1*, *polyubiquitin* (*UbII*) and *ferritin heavy chain* (*Fth*) [37]. To date, all of the above genes where this was studied (*ERNI*, *Churchill*, *Sox3*, *UbII* and *Calfacilitin*) turned out to be regulated by FGF8, but not by BMP antagonists.

Here we describe the remaining three genes isolated from the screen. These encode the neurotrophin receptor *TrkC* [38] and two previously uncharacterized proteins, which we name *Obelix* and *Asterix*. Like all other genes from the screen, these three are expressed in the prospective embryonic neural plate of the normal embryo and are induced within 5 hours of a Hensen's node graft into the area opaca. *Asterix* is also induced by FGF8 under the same conditions. However *Obelix*, which encodes a putative RNA-binding protein and *TrkC* are not regulated by FGF, by BMP antagonists or by other factors implicated in neural induction in previous studies. These observations lead us to suggest that FGF is the major, but not the only, signal involved in the early steps of neural induction, and that other signals remain to be identified. Examination of the timing of induction of all of these response genes after a node graft reveal that they are deployed in three “epochs”, or temporal waves, both in the neural induction assay and during normal development. Genes induced within 3 hours of a node graft (*ERNI*, *Sox3*, *Calfacilitin* and *Geminin*) are normally expressed in the epiblast of pre-primitive-streak stage embryos. Other genes are induced within 5 hours of grafting Hensen's node (*Churchill*, *UbII*, *Dad1*, *Fth*, *TrkC*, *Asterix* and *Obelix*) – in normal embryos, these start to be expressed in the prospective neural plate from the late primitive streak stage. A third “epoch” comprises genes induced after about 12 hours of a node graft such as *Sox2* and *BERT* [39]. In normal embryos these genes start to be expressed in the very early neural plate when the axial mesoderm starts to form, at stage 4^+^-5. Of this list of genes, three are induced by unidentified signals from the node: *TrkC*, *Obelix* (this study) and *BERT* [39]. Since these genes belong to different “epochs”, we suggest that at least two further signals involved in neural induction remain to be identified. The present results help to define assays to find these signals.

## Results

### Isolation of two novel response genes to neural induction

An initial survey of 5,000 plaques from the original differential screen for early responses to signals from a graft of Hensen's node [35] identified 49 clones showing differential expression between the induced and uninduced libraries. Of these, 10 corresponded either to already known genes (*Sox3* and *TrkC*, as determined by Southern blotting) or hybridized with cDNA probes from the quail node. The remaining 39 clones were analyzed and found to correspond to two different genes, initially designated “R1”, represented 37 times and “R2”, represented twice. R1 was subsequently named *ERNI* [35], [39]. The screen was then extended to 500,000 plaques, which identified other differentially expressed genes including novel transcripts *Churchill* [36], *Calfacilitin* (Papanayotou et al., in preparation) and others that had been studied in other contexts *Dad1*, *Fth* and *UbII* [37]. We start here by describing briefly the two novel genes: R2 (*Asterix*) and a clone from the extended screen, initially designated b1 (*Obelix*). Then we study the normal expression and the regulation of these genes and of *TrkC* by signals from the organizer.

#### a. Characterization of *Obelix*

**Figure 1:**
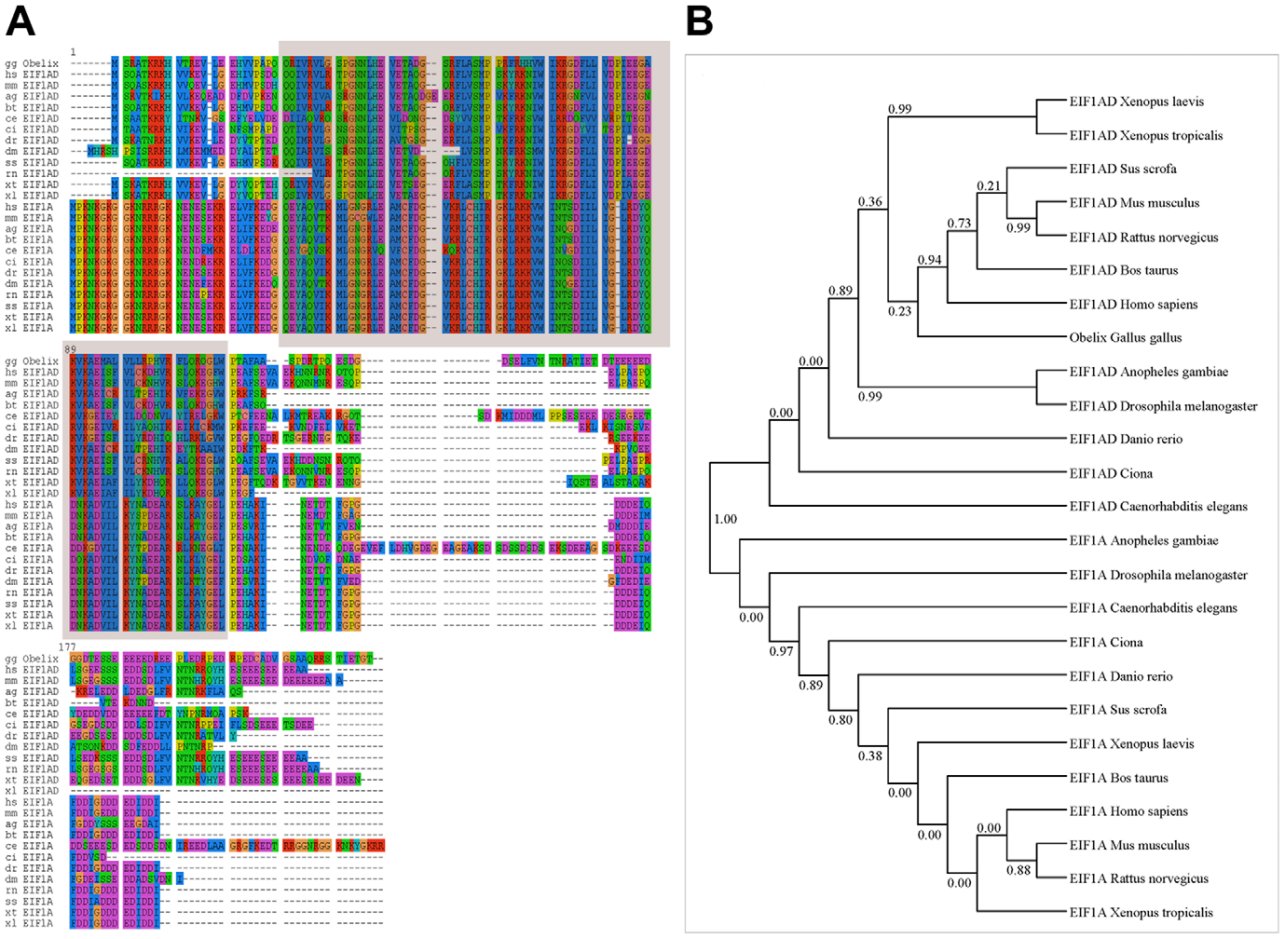
Molecular characterization of Obelix. **A.** Sequence alignment of Obelix protein (AY103477) with ESTs for EIF1A-related proteins from several species. Residues are displayed in different colours based on different aminoacid families and degree of homology is represented by conservation of these sites. Conserved OB-like domain is shown as a block in the alignment. Species are abbreviated as follows: ag, *Anopheles* mosquito (BM594550); bt, cow (BF043073); ce, *C. elegans* (AV203381); ci, *Ciona* (AV841463); dm, *Drosophila melanogaster* (BE977318); dr, zebrafish (BM859434); hs, human (BG149615); mm, mouse (BI103120); ss, pig (BG610103); rn, rat (BF420639); xl, *Xenopus laevis* (BG730245); xt, *Xenopus tropicalis* (AL637659). **B.** Phylogenetic tree with bootstrap values comparing the full-length sequences of Obelix in a variety of species, showing that eIF1A and Obelix segregate into two distinct sub-classes of OB-containing proteins. The LG model was used to construct the tree and bootstrap values were calculated from 1000 replicates.

The b1 clone was used to screen a stage 2–4 cDNA library; four independent clones were analyzed. The sequence is 781 nucleotides long, containing a predicted 564 nucleotide open reading frame (188 aminoacids), 54 5′ untranslated (UTR) nucleotides and 72 nucleotides of 3′ UTR. Blast searches using the aminoacid sequence reveal a conserved domain (aminoacids 25–103) within which is a region (aminoacids 33–96) similar to the oligonucleotide/oligosaccharide-binding (OB) domain of the Translation Initiation Factor eIF1A, responsible for the RNA-binding properties of this protein [40] (Fig. 1A). Because of this, and its predicted globular shape, the protein encoded by the b1 gene is designated Obelix (GenBank accession number AY103477).

Phylogenetic bootstrap analysis using SeaView 4.2.12 [41] reveals that Obelix clusters closer to sequences from human (NM_032325), mouse (NM_027236), Drosophila (NM_164564) and other species (currently labelled as EIF1AD) than with true EIF1A from human, mouse or Drosophila (NP_001403, XP_110004 and AAF44294, respectively) and their orthologues in other species (Fig. 1B). Therefore Obelix and its related proteins are distinct from eIF1A. The predicted structure of the OB domain of Obelix appears very similar to that of eIF1A, but contains an extra sheet structure between sheets β3 and β4. In eIF1A, strands β3 and β4 are connected instead by a long loop, which is the most variable portion of the OB-fold, but usually contains a helix [40].

**Figure 2:**
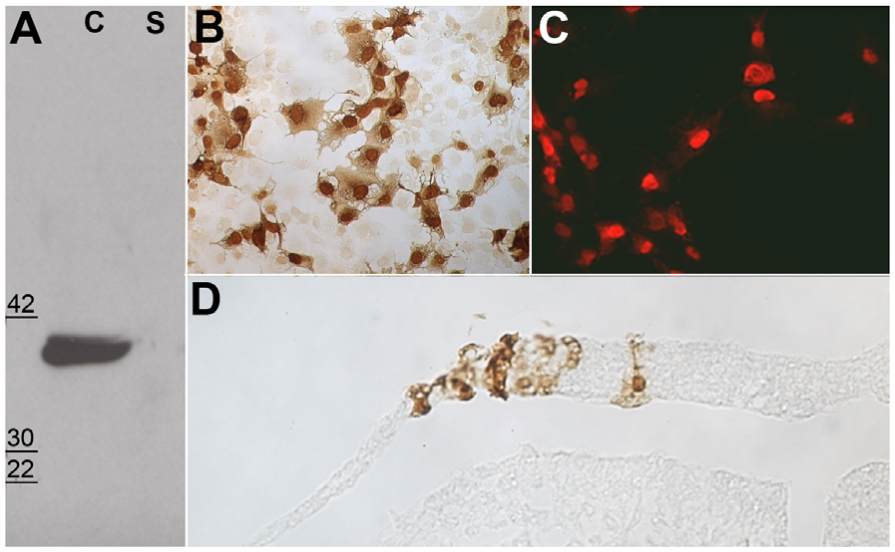
Obelix is intracellular and localizes to the nucleus. **A.** Obelix protein can be retrieved from cell extracts (C) but not from the supernatant (S) of transfected COS-1 cells, and detected by Western blotting. **B–D.** Nuclear localization of Myc-tagged Obelix protein can be seen in transfected COS-1 cells (B, C) as well as in the neural plate of a chick embryo (D). In B and D the anti-Myc antibody is revealed by peroxidase staining with diaminobenzidine; in C the signal is revealed with Cy3-coupled anti-mouse antibody.

To gain insight into the intracellular location of Obelix, a myc-tagged version was transfected into COS-1 cells, and the presence of Obelix protein in cell lysates and medium was assayed by Western blotting. Obelix protein was detected in the cell lysate (C, Fig. 2A) but not in the supernatant (S, Fig. 2A), suggesting a cellular protein that is not secreted. Immunostaining of transfected COS-1 cells or transfected chick embryo epiblast revealed a predominantly nuclear localization (Fig. 2 B–D).

#### b. Characterization of *Asterix*

**Figure 3:**
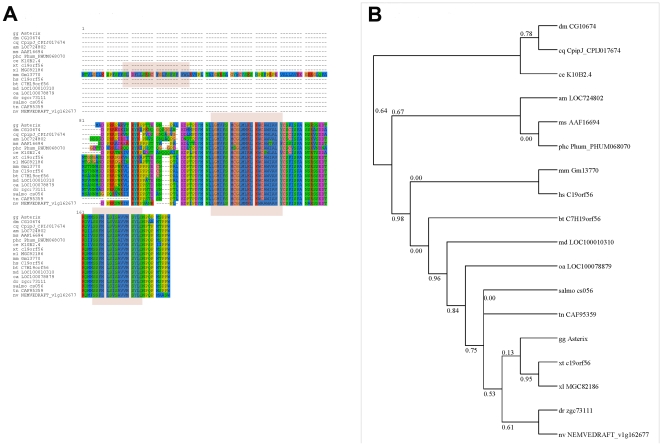
Molecular characterisation of Asterix. **A.** Sequence alignment of Asterix protein with related proteins in other species. Residues are displayed in different colours based on different aminoacid families and degree of homology is represented by conservation of these sites. Predicted transmembrane domains are outlined as blocks. Abbreviations for the following organisms appear in the alignment in the following order from top to bottom: Chick (gg) Asterix; Drosophila melanogaster (dm) protein (CG10674); Culex quinquefasciatus (cq) hypothetical protein (CpipJ_CPIJ017674); Apis mellifera (am) protein (LOC724802); Manduca sexta (ms) unknown protein (AAF16694); Pediculus humanus corporis (phc) hypothetical protein (Phum_PHUM068070); Caenorhabditis elegans (ce) hypothetical protein (K10B2.4); Xenopus tropicalis (xt) protein (c19orf56); Xenopus laevis (xl) protein (MGC82186); Mus musculus (mm) predicted gene 2573 (Gm13770); Homo sapiens (hs) protein (c19orf56); Bos taurus (bt) c19orf56 ortholog (C7H19orf56); Monodelphis domestica (md) similar to CGI-140 protein (LOC100010310); Ornithorhynchus anatinus (oa) hypothetical protein (LOC100078879); Danio rerio (dr) hypothetical protein (zgc:73111); Salmo salar (salmo) UPF0139 membrane protein C19orf56 homolog (cs056); Tetraodon nigroviridis (tn) unnamed protein (CAF95359); Nematostella vectensis (nv) hypothetical protein (NEMVEDRAFT_v1g162677). **B.** Phylogenetic tree with bootstrap values comparing the full-length sequence of Asterix with homologues from other species. The LG model was used to construct the tree and bootstrap values were calculated from 1000 replicates.

Blastn searches using the R2 sequence identify a predicted transcript, ENSGALT00000012756 and an EST sequence, ChEST53I6 (Unigene Gga. 10371), containing 5 predicted exons from gene ENSGALG00000007858. However, the R2 cDNA sequence continues upstream from the predicted transcript and EST (Fig. 3). The predicted protein product belongs to an Uncharacterized Protein Family designated UPF0139, also designated CGI140 or c19orf56 in humans because the predicted open reading frame appears on chromosome 19. It appears to be very highly conserved across all vertebrates as well as invertebrates (Fig. 3). Because of the smaller size of the predicted R2 protein as compared to Obelix and their co-expression we designate R2 as Asterix (Genbank accession number HQ184923).

### Expression of *Obelix* and *Asterix* during development

**Figure 4:**
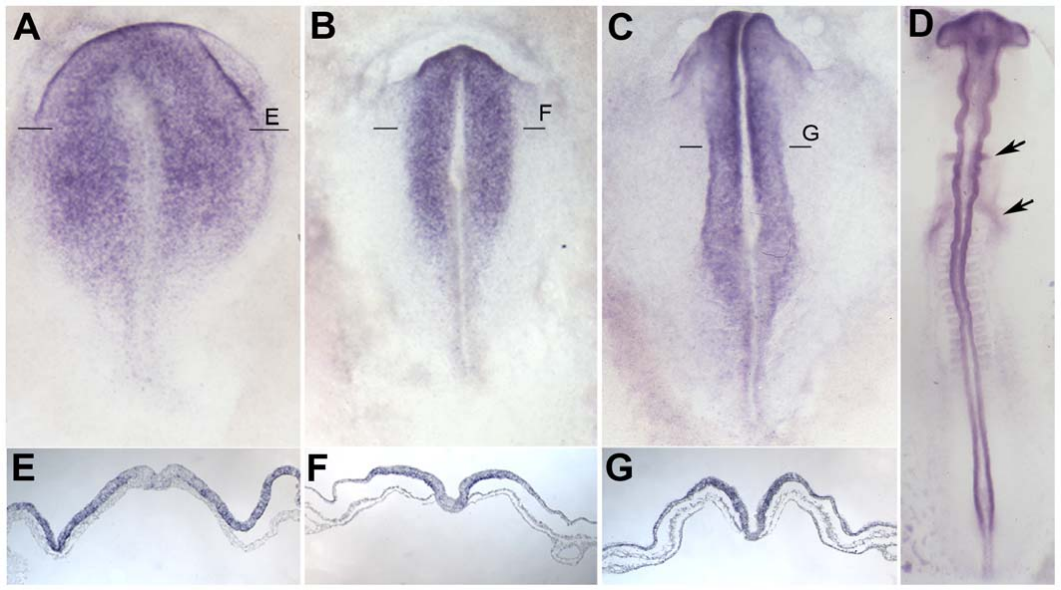
*Obelix* expression during early development. Expression of *Obelix* by in situ hybridization at stages 3+ (A), 5 (B), 7 (C) and 11 (D). E–G are sections through the levels shown in A–C. Expression is localized in the neural plate, neural tube and their derivatives.

Both *Obelix* and *Asterix* were identified from a screen for early responses to signals from a grafted organizer (Hensen's node). If these genes are indeed responses to neural induction, they should also be expressed in the early neural plate of normal embryos at appropriate stages. To test this, whole mount in situ hybridization was performed on early chick embryos. *Obelix* transcripts are first detected at the mid- to late-primitive-streak stage (stage 3^+^), initially in a region of the area pellucida a little broader than the future neural plate (Fig. 4 A, E). Expression quickly becomes confined to the neural plate (Fig. 4 B, F) where it remains until at least stage 14, including streams of neural crest cells migrating away from the neural tube (Fig. 4 D).

**Figure 5:**
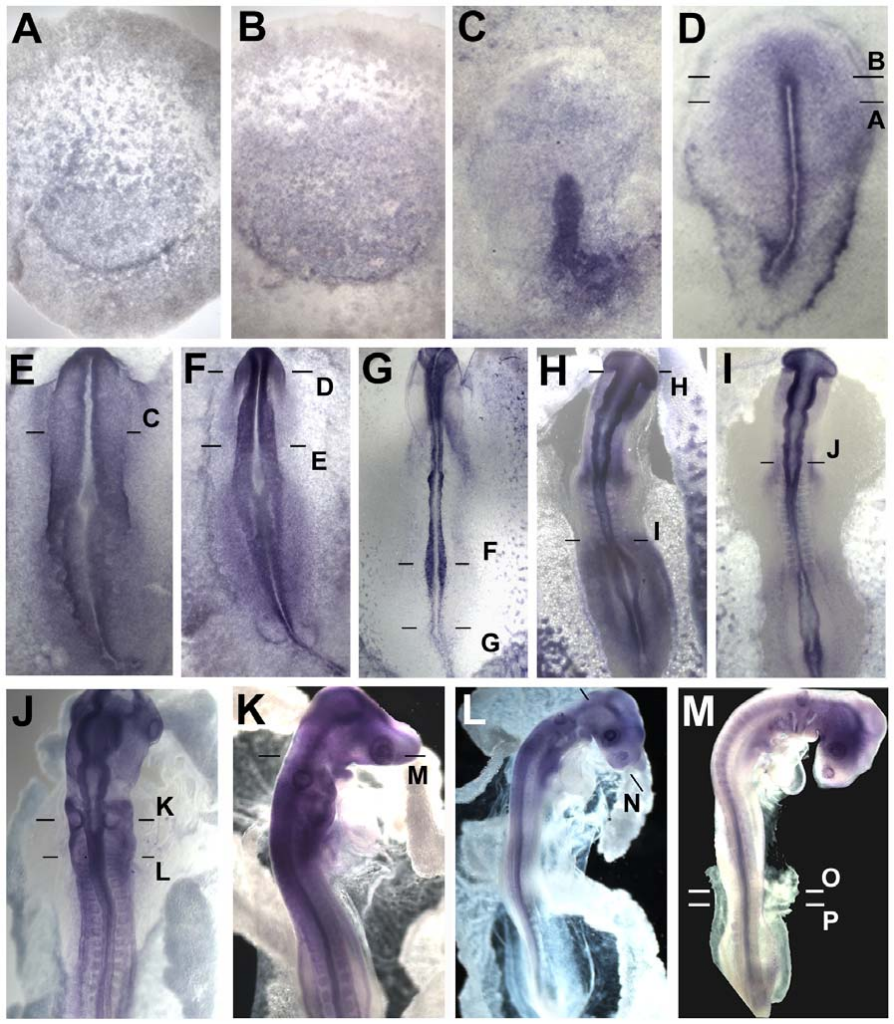
Expression of *Asterix* during development. Embryos at stages XI (A), XII (B), 3 (C), 4+ (D), 6 (E), 7 (F), 9 (G), 10 (H), 11 (I), 14 (J), 16 (K), 17 (L) and 18 (M) are shown. The horizontal lines and letters refer to the levels at which sections in Fig. 4 were taken.

**Figure 6:**
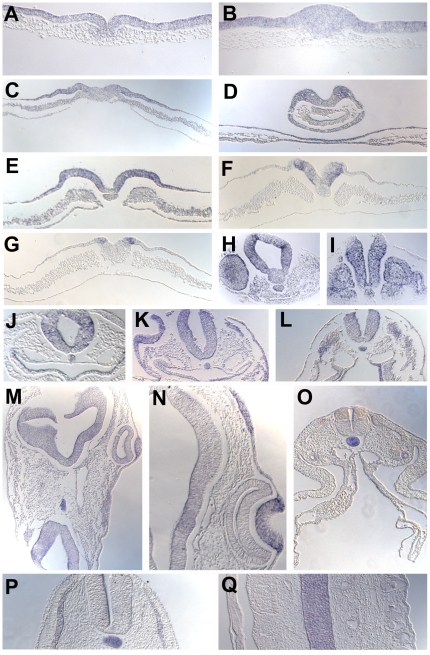
Expression of *Asterix* during development (continued). Sections through embryos at stages 4+−18, at the levels indicated in Fig. 3. Q shows a coronal section through an embryo at stage 16, showing expression in the notochord.

*Asterix* expression is first detected very weakly in the hypoblast and Koller's sickle at pre-primitive streak stages [42]; stages XI-XIII; Fig. 5 A–B). During primitive streak formation it is expressed in the streak itself (Fig. 5 C). From stage 4 (Fig. 5 D, Fig. 6 A, B) expression is seen in the node, the lips of the streak and the epiblast in the middle of the area pellucida but is absent from more peripheral regions (future epidermis and extraembryonic ectoderm). By the start of neurulation (stage 7) expression becomes progressively concentrated in the neural plate (Fig. 5 E–H, Fig. 6 C–I), neural tube (Fig. 5 I–K, Fig. 6 J–M) and sensory placodes including lens, otic and olfactory placodes (Fig. 5 J–M, Fig. 6 K–N). From about stage 16 expression starts to decrease in the nervous system to become concentrated mainly in the notochord (Fig. 5 M, Fig. 6 O–Q), as well as remaining in the sensory placodes. Some expression is also seen in somites (eg. Fig. 5 H, Fig. 6 I) and persists in the myotome at later stages (Fig. 6 P).

In conclusion, both *Obelix* and *Asterix* are expressed in the developing nervous system. They appear in the prospective neural plate during gastrulation (stage 3^+^) and remain expressed in the neural plate, neural tube, neural crest (*Obelix*) and placodes (*Asterix*) at least until stage 14.

### *TrkC* expression during development

**Figure 7:**
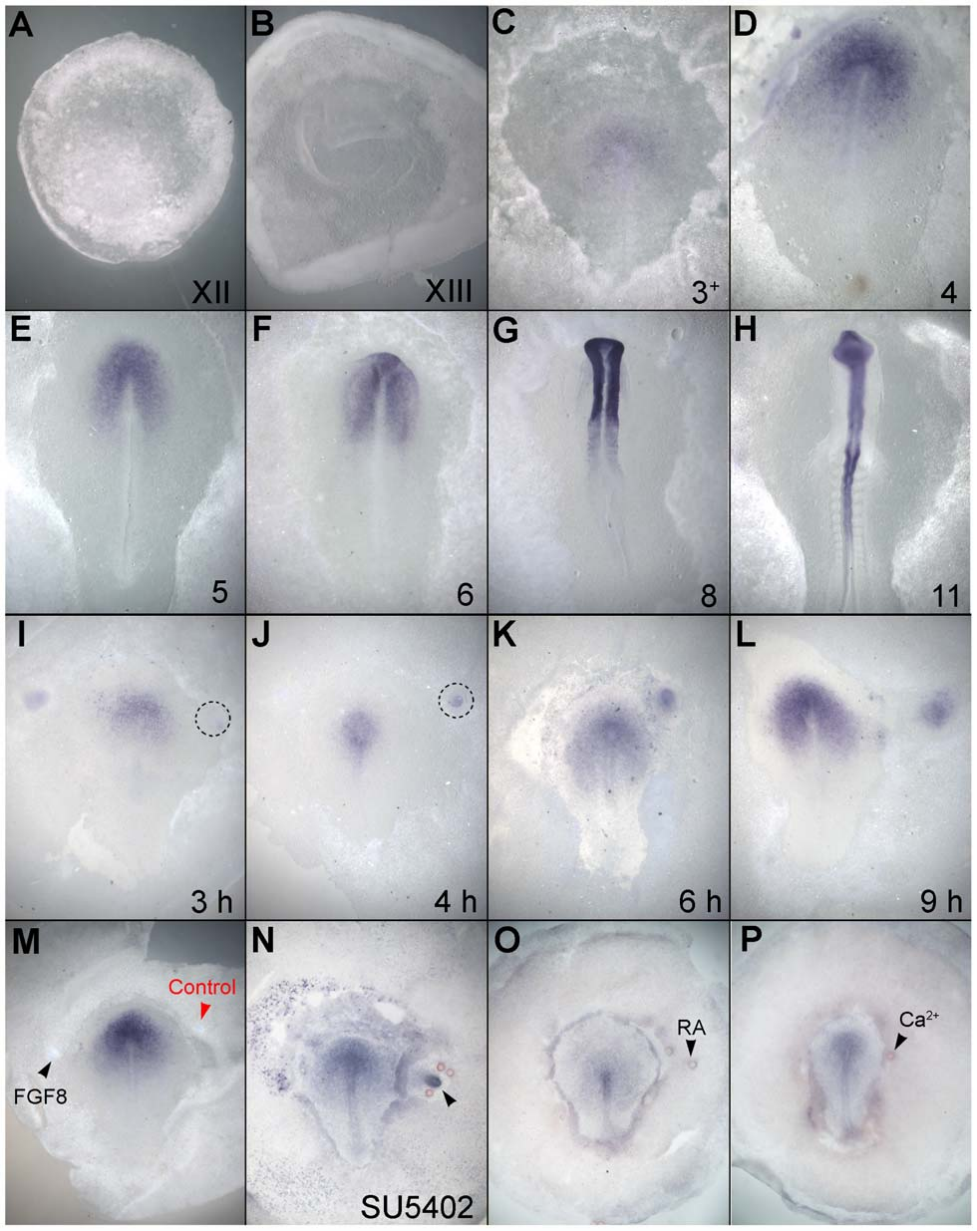
Expression and regulation of *TrkC*. **A–H.** *TrkC* expression during early chick development. The stage of development is indicated on the lower right of each panel. Expression begins at mid-primitive streak stage (stage 3^+^; C) and intensifies in the neural plate thereafter (E–H). **I–L.** Time-course of induction of *TrkC* by a graft of Hensen's node. Induction begins about 4 hours after grafting (J) and becomes strong after about 6 hours (K, L). **M.** *TrkC* is not induced by FGF. Embryo shown 6 hours after implantation of a bead soaked in FGF8b (black arrow) and a control bead (red arrow). **N.** Induction of *TrkC* by the organizer does not require FGF signals. Embryo shown 6 hours after co-transplantantation of a Hensen's node and three beads soaked in SU5402. Induction of *TrkC* is not inhibited (arrow). **O.** *TrkC* is not induced by retinoic acid (arrow). **P.** *TrkC* is not induced by a bead soaked in ionomycin to increase intracellular Calcium (Ca^2+^; arrow).

A third gene identified by our screen for early responses to a grafted node encodes the neurotrophin receptor, *TrkC*. Its expression has been described during quail development but not in sufficient detail to determine a precise time course during neural induction [38], [43]–[45]. We therefore studied its expression by in situ hybridization in chick embryos between pre-streak and neural plate stages (Fig. 7 A–H). Transcripts are first detected close to Hensen's node at stage 3^+^ (Fig. 7 C–D) from where expression expands to the forming neural plate between stages 4–7 (Fig. 7 E–G). Thereafter it remains expressed almost throughout the neural plate except in the most caudal domains and in sub-regions of the hindbrain (Fig. 7 G, H) (see also [38], [45]).

### Time-course of induction of *TrkC*, *Obelix* and *Asterix* by the organizer

**Figure 8:**
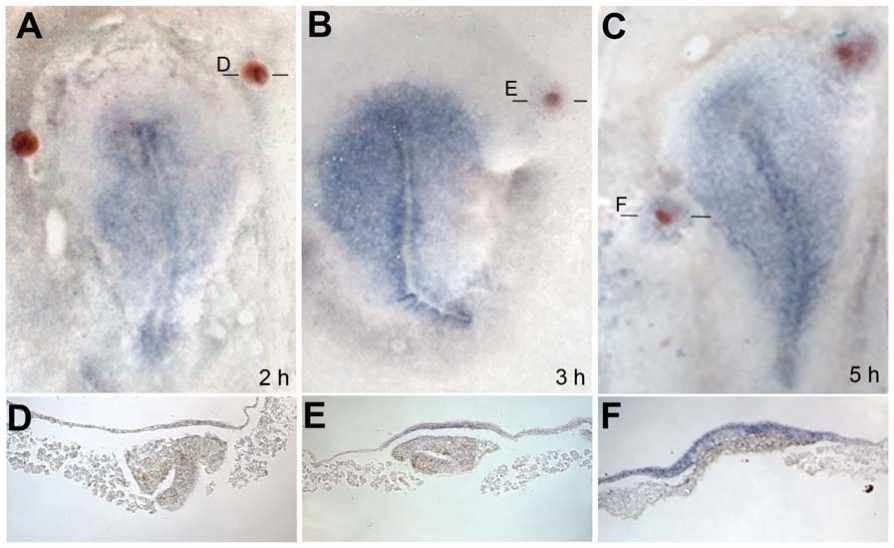
Time-course of induction of *Obelix* by Hensen's node. Time-course of induction by grafts of a quail node into a chick host. No induction is seen at 2 hours (A), weak induction starts at 3 hours (B) and robust induction is seen by 5 hours (C). D–F are sections through the grafted regions of the embryos in A–C at the levels indicated. Quail donor cells are stained brick-red by QCPN antibody and *Obelix* mRNA in purple/blue.

**Figure 9:**
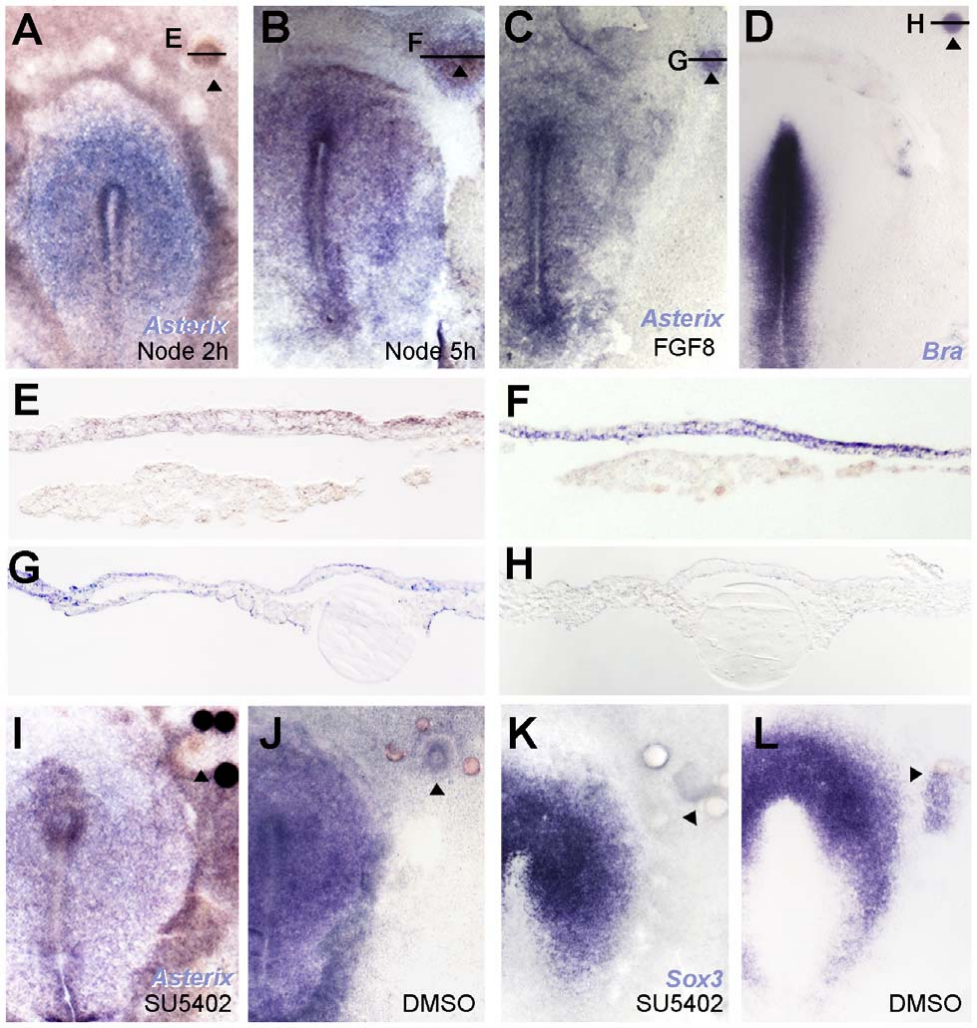
Regulation of *Asterix* by Hensen's node and peptide factors. Grafts of Hensen's node do not induce *Asterix* after 2 hours (A, E), but do induce it after 5 hours (B, F). This is mimicked by grafts of heparin beads soaked in FGF8, which induce *Asterix* (C, G) but not *Brachyury* (D, H). When a node is grafted together with beads soaked in the FGF inhibitor SU5402, *Asterix* induction is blocked (I), as is induction of the early pre-neural marker *Sox3* (K). Grafts of the node together with the vehicle DMSO do not affect induction of either marker (J for *Asterix*, L for *Sox3*). Note that some probes attach non-specifically to some types of beads and to COS cell pellets (eg. panels H, I).

To confirm that *TrkC*, *Obelix* and *Asterix* are indeed early response genes to signals from the organizer, their induction by Hensen's node grafts was studied in time-course. No induction is seen 2 hours after a node graft into the area opaca of HH3+/4 host embryos (*Obelix*: 0/4; Fig. 8 A, D; *Asterix:* 0/6; Fig. 9 A, E). At 3 hours, *Obelix* and *TrkC* are very weakly induced in a minority of embryos (*Obelix*: 4/13, Fig. 8 B, E; *TrkC*: 6/8 Fig. 7I) and *Asterix* not at all (0/7). A short time later, all genes are strongly induced: *Obelix* induction appears 5 hours after the node graft (18/19; Fig. 8 C, F), and *Asterix* and *TrkC* by 4–5 hours (*TrkC*: 4 hours: 3/3; 5 hours: 3/3; 6 hours: 5/5; Fig. 7 J, K; *Asterix:* 4 hours: 7/9; 5 hours: 7/8; Fig. 9 B, F). Together with the findings that all three genes are normally expressed in the prospective and early neural plate, these findings confirm *TrkC*, *Asterix* and *Obelix* as early responses to neural inducing signals from the organizer, Hensen's node.

### Regulation of *Asterix*, *Obelix* and *TrkC* by secreted factors

Next, we sought to determine whether any known secreted factors implicated in neural induction or expressed in the organizer can mimic the ability of the node to induce *Asterix*. Unlike BMP- and Wnt-inhibitors, FGF8-soaked heparin beads induce *Asterix* expression in the adjacent epiblast within 5 hours (2–3 h: 2/16; 4–5 h: 12/15; 16–18 h: 7/9; Fig. 9 C, G) without inducing the mesodermal marker *Brachyury* (0/10; Fig. 9 D, H).To test whether FGF signalling from the organizer is required for Asterix induction, a node was transplanted together with beads soaked in SU5402, an inhibitor of the FGF receptor [46], into the extraembryonic region. This completely abolishes *Asterix* induction (0/8; Fig. 9 I), unlike control DMSO-soaked beads (4/4; Fig. 9 J). SU5402 also inhibits induction of *Sox3* by a grafted node (0/6; Fig. 9 K, 4/4 control, Fig. 9 L), as previously described [35]. In conclusion, FGF8 mimics the ability of the node to induce *Asterix* within 5 hours and FGF activity is necessary for its induction by the node.

**Figure 10:**
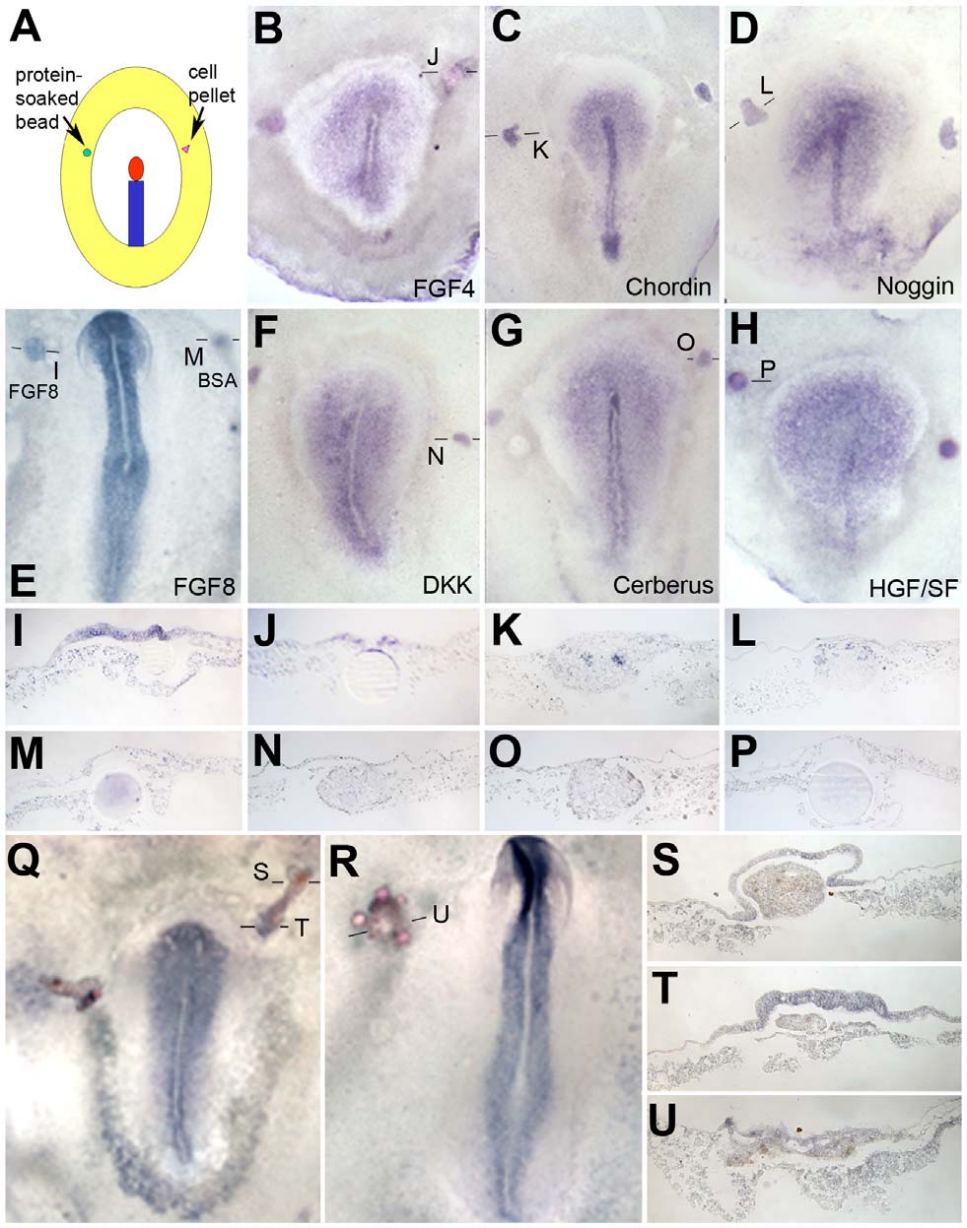
Regulation of *Obelix* by various secreted factors. **A–P**. The ability of various peptide factors to induce *Obelix* expression was tested by local application of beads soaked in the protein or pellets of COS-1 cells transfected with a construct encoding the factor into the area opaca of a host embryo (A). Examples of FGF4 beads (B), Chordin (C) and Noggin (D) cells, FGF8/control beads (E), Dickkopf (F), Cerberus (G) cells and HGF/SF beads (H) are shown. I–P show sections through the grafted region of the embryos in B–H at the levels indicated. **Q–U**. Co-transplantation of a quail Hensen's node with beads soaked in the FGF inhibitor SU5402 has little or no effect: Obelix is still induced (Q–U). Q shows a grafted embryo fixed after 6 hours, and R is an example of an embryo grown overnight after the graft. S–U are sections through these embryos at the levels indicated in Q and R. Quail cells are stained with QCPN (brown). Note that some probes attach non-specifically to some types of beads and to COS cell pellets (eg. panels K–H).

Rather different results are obtained for *TrkC* and *Obelix*. FGF8 does not induce *TrkC* expression at all (0/11 after 6 hours, 0/12 after 14 hours; Fig. 7 M). FGF4 or FGF8-coated beads do induce *Obelix* (6/14 and 11/15, respectively; Fig. 10 B, E, I, J). However, in contrast with Hensen's node grafts, induction by either factor is weak, localized to the immediate vicinity of the bead and only seen in a subset of embryos (17/29, 58%). We also tested many other candidate factors, none of which induces either gene: retinoic acid (*Obelix:* 0/11; *TrkC*: 0/4, Fig. 7 O), ionomycin to increase intracellular Calcium (*Obelix*: 0/9; *TrkC*: 0/4, Fig. 7 P), Chordin (*Obelix*: 0/8; Fig. 10 C), Noggin (*Obelix*: 0/6; Fig. 10 D, L), the Wnt antagonist Dkk1 (*Obelix*: 0/10; Fig. 10 F, N), Cerberus (*Obelix*: 0/12; Fig. 10 G, O), HGF/SF (*Obelix*: 0/10; Fig. 10 H, P) and Somatostatin (*TrkC*: 0/8; *Obelix*: 0/15).

These results suggest that FGFs induce *Obelix*, but only weakly and not in all cases and that *TrkC* is not induced by FGF at all. To test whether FGF signalling from the node is required for *TrkC* and *Obelix* induction, we transplanted the organizer together with SU5402-coated beads. Both *TrkC* (6/6; Fig. 7N) and *Obelix* (4/5; Fig. 10 Q–U) are induced even when FGF signalling is inhibited. This is in contrast to *ERNI, Sox3*, *Churchill* and *Sox2* induction, all of which require FGF activity in the same assay [35], [36].

In conclusion, the three genes differ in the extent to which they are inducible by FGF: FGF is both necessary and sufficient to induce *Asterix*, sufficient but not necessary to induce *Obelix* and neither sufficient nor necessary for induction of *TrkC*. A plausible interpretation is that *Obelix* may be induced by a factor other than FGF which acts through the same pathway(s), such as IGF or PDGF, whereas TrkC is likely to be induced by factor(s) acting through other pathways.

## Discussion

### The genes: Obelix, Asterix and TrkC

Our screen for early responses to neural induction signals was designed to identify new markers for the earliest responses to neural inducing signals, upstream of BMP inhibition, which could in turn be used to identify the missing signals. Previously we had reported on 7 genes: *ERNI*, *Churchill* and *Calfacilitin*, *Dad1*, *UbII*, *Fth* and *Sox3*, which led to the conclusion that FGF is a crucial early signal. Here we describe the remaining 3 genes from the screen: two that had not previously been studied and one known to encode TrkC, the receptor for the neurotrophin NT-3 but not hitherto associated with neural induction. Although a functional study of these genes and of their involvement in the neural induction process is not the purpose of this study, we begin this section with a brief discussion of their molecular properties before considering their regulation by the organizer, their position in the neural induction hierarchy and their regulation by secreted signals.

#### a. Obelix defines a new family of putative RNA-binding OB-domain proteins

Obelix displays some sequence similarity with eIF1A, a small, acidic protein and one of the most conserved eukaryotic translation initiation factors [47], [48]. The region of greatest conservation between eukaryotic (eIF1A) and the shorter prokaryotic (IF1) translation initiation factors [49]–[51] encompasses the oligonucleotide/oligosaccharide-binding (OB) fold, which in human eIF1A is responsible for binding and scanning the mRNA [40], [52]. Different oligonucleotide- and oligosaccharide-binding proteins share this OB fold motif [53], comprising a five-stranded β-sheet, coiled to form a closed β-barrel, which has been described as a “five-stranded Greek key β barrel” [54], with strands proceeding from the amino- to the carboxy-terminus. Obelix does not show any identity to eIF1A in regions outside the OB-domain, suggesting that its function may involve RNA binding but is unlikely to act as an elongation initiation factor, which is also consistent with the finding that this is a predominantly nuclear protein. Phylogenetic comparisons of related sequences from a variety of organisms (Fig. 1B) reveal that the major homology between Obelix and other proteins (including eIF1A) is in the oligosaccharide/oligonucleotide-binding (OB) domain. Obelix-related proteins in human, mouse and Drosophila cluster together with Obelix but not with human, mouse and fruitfly eIF1A, suggesting that eIF1A- and Obelix-related proteins represent different sub-families within a larger group of OB-domain-containing proteins.

#### b. Asterix – a novel protein of unknown function

It is difficult to speculate on a possible function of Asterix, or even to determine its most likely structure. Different subcellular and structure prediction programs predict different properties. TMMHMM Server 2.0 predicts two transmembrane domains, one between aminoacids 35–57 and the second between aminoacids 77–92, with the intervening region predicted as intracellular and the amino- and carboxy-termini as extracellular. In contrast PSORTII (k-NN prediction) predicts a nuclear protein (43.5% probability) or mitochondrial localization (39.1%). The same programs applied to possible Myc-, FLAG- or His-tagged versions of the protein make even more ambiguous predictions and we therefore decided that this would not be a reliable method for determining subcellular localization of this protein.

Asterix appears to be as well conserved as Obelix. Genomic and other databases suggest Asterix as a founder member of an uncharacterized protein family (UPF0139), identified in humans as c19orf56 (a predicted open reading frame on Chromosome 19), annotated in humans and other species as CGI140 with the prediction that this is integral to the membrane. Despite its conservation, there are no functional or expression data available in the literature.

#### c. TrkC expression in early neural plate development

Unlike the above two genes, TrkC is a very well studied membrane protein encoding the receptor for the neurotrophin NT-3. Its expression had been described in quail [38], [43]–[45] and a few other species but not implicated as an early response to neural induction. Despite earlier reports [43], [55] that *NT-3* mRNA can be detected by RT-PCR from before primitive streak formation, we were unable to detect it by in situ hybridization at these early stages (data not shown). Therefore if TrkC has a function at these very early stages it either involves interactions with a different ligand or a ligand-independent mechanism.

### A temporal hierarchy of responses to organizer signals

The screen for early responses to neural induction was conducted using cells from the extraembryonic epiblast of the area opaca, to ensure that the events to be studied relate to the initial exposure of cells to inducing signals. Therefore it is important to determine that the genes identified from this screen must also be expressed in the normal prospective embryonic neural plate at some stage in its development. This was indeed the case for all genes isolated to date, *ERNI*, *Churchill*, *Dad1*, *polyubiquitin*, *Fth*, *calfaclitin* and *Sox3* [35]–[37], [39], [56], [57] and is also true for *Asterix*, *Obelix and TrkC*.

**Figure 11:**
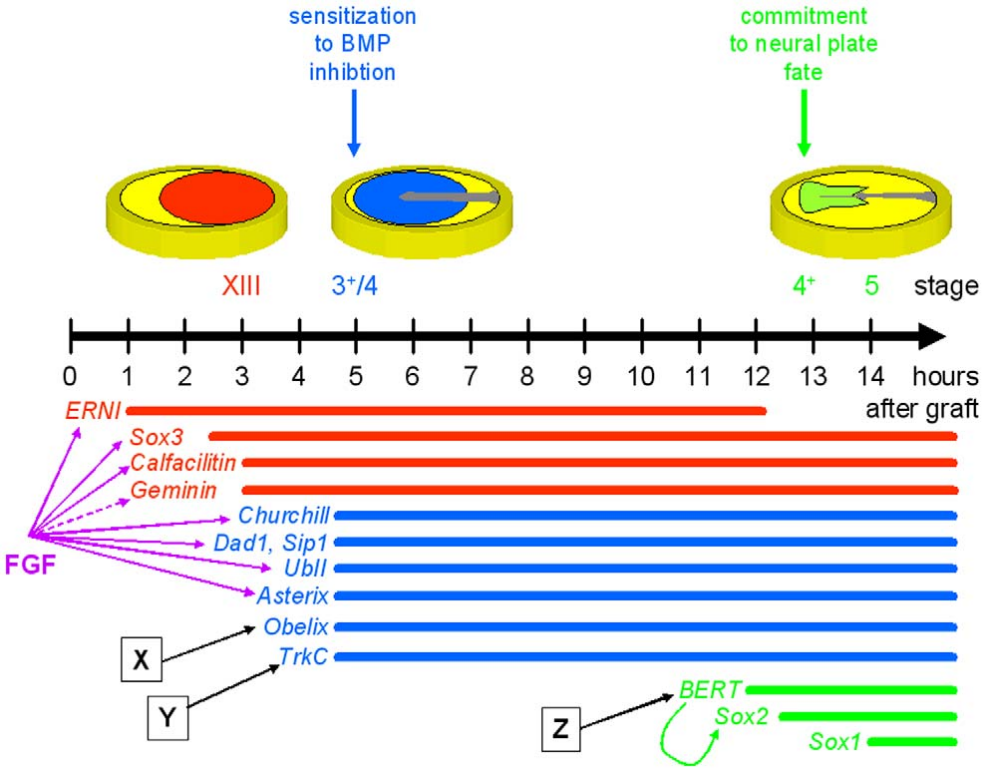
Time-course of markers during neural induction and their regulation by signals. Temporal hierarchy of deployment of 13 early neural markers. The colored lines at the bottom of the figure represent the period of expression of these genes, in relation to the time at which they are induced following a graft of Hensen's node into the area opaca (in hours on the scale above) and in relation to the stage of normal embryos at which they are expressed (stage shown above the time line). The diagrams above these stages schematize the domains of expression. The genes fall into three “epochs”: those induced by a node within 3 hours start to be expressed in normal embryos before streak formation (red). Those induced by a node in 4–5 hours begin their expression at the mid- to late-primitive streak stage (blue) and those that are induced by a node at 12–13 hours do not begin their expression until the end of gastrulation, in the forming neural plate (green).

With 10 genes now known to be regulated within 5 hours of exposure to an organizer and with data on their normal expression, it now becomes apparent that even within these initial 5 hours, they fall into two distinct classes. Several of them start to be expressed at the mid-primitive streak stage (3–3^+^). This group (“streak group”; blue in Fig. 11) includes *Churchill*, *Dad1*, *polyubiquitin* and *Fth.* A few genes (*ERNI*, *Sox3* and *calfacilitin*) start to be expressed even before primitive streak formation (“pre-streak group”; red in Fig. 11). *TrkC* and *Obelix* belong to the streak group, as their expression starts at the mid-/late primitive streak stage. Although *Asterix* expression begins before primitive streak formation, transcripts are only seen in the hypoblast at these early stages; expression in the epiblast only begins at stage 3–3^+^ and it therefore also belongs to the streak group. Like most other genes isolated from the screen, all 3 genes studied here remain expressed in the neural plate and neural tube at later stages of development.

The genes are also not all induced at the same time by a grafted organizer. *ERNI* is induced within 1–2 hours, *Sox3* and *calfacilitin* at 2–3 hours, and the remaining genes are only induced robustly 4–5 hours after grafting. The order in which these genes are induced mirrors the sequence of stages at which expression of the same gene is first seen in the normal embryo (see above): genes induced within less than 3 hours of grafting are expressed in the embryonic epiblast before gastrulation (*ERNI*, *Sox3* and *calfacilitin*) (“pre-streak group”) whereas those that require more than 3 hours start to be expressed only at around the mid-primitive streak stage (“streak group”). The timing of *Asterix*, *Obelix* and *TrkC* induction by a grafted node fits well into this scheme. All three are induced weakly after 3 hours and robustly by 4–5 hours after grafting, consistent with their expression in the normal embryonic epiblast from primitive streak stages onwards. Thus, the genes isolated from the screen reveal two discrete “epochs” of gene expresssion associated with neural induction by an organizer graft, and these parallel similar phases of gene expression during normal neural plate development. A third distinct phase is marked by the expression of *Sox2* and *BERT*, which appear after 12–13 hours following a node graft and at stages 4^+^–5 in the normal neural plate (early neurulation).

### Tissues and factors responsible for induction of different genes

Unless exposed to node signals for at least 5 hours, chick epiblast cells cannot respond to BMP inhibitors [28]. Thus, signals other than node-derived BMP antagonists are required to initiate the neural induction cascade and to sensitize cells to BMP inhibition. Studies on the regulation of *ERNI* and *Churchill* pointed to FGF as a crucial upstream signal initiating neural induction [35], [36], [39]. Hensen's node expresses FGF8 at the appropriate stages of development [29]. However, those genes that are induced in less than 3 hours and which are normally expressed in the epiblast before streak formation (*ERNI*, *Sox3*, *Calfacilitin* and *Geminin*) cannot be induced by the node during normal development, since this structure only appears at a later stage. At these early stages, the most likely source of FGF is the hypoblast, an extraembryonic tissue equivalent to the Anterior Visceral Endoderm of the mouse [58], [59]. Indeed, grafts of hypoblast cells from a pre-primitive-streak stage donor into the area opaca of a primitive-streak stage host do induce expression of *ERNI* and *Sox3* [35], [60], but they cannot induce expression of later pre-neural or neural markers. These results make it likely that during normal development, neural induction is initiated by FGF emanating from the hypoblast, which induces expression of very early genes in the cascade. In addition to *ERNI* and *Sox3*, the hypoblast can also induce *Cyp26A1* and *Otx2* [60], but neither of these can be induced by FGF. Retinoic Acid (RA) appears to be involved in inducing both of these, either alone or in combination of with other factors [60].

Despite the conclusion that during normal development the induction cascade may be initiated by signals from the hypoblast, it is clearly the case that a graft of Hensen's node is sufficient to induce a well patterned nervous system even in regions that have never been exposed to the hypoblast [33], [34], [61]–[63]. This can be explained by the findings that the node expresses both FGF8 [29] and Retinoids [64], [65] at the stage at which it is capable of inducing an ectopic neural plate when grafted into the area opaca. Thus, organizer grafts can induce the nervous system because they also contain signals which during normal development are provided by other tissues – the target genes of this induction are those which we now define as the “pre-streak group” (red in Fig. 11).

*ERNI*, *Sox3*, *Calfacilitin*, *Polyubiquitin*, *Dad1, Churchill* and *Asterix*, can all be induced by FGF8. For all of these, induction by a node graft is abolished by SU5402, a pharmacological inhibitor of the FGF receptor, revealing that FGF signalling from the node is required for induction by the organizer. Indeed, a requirement for FGF in neural induction in addition to BMP inhibition was suggested by experiments in Xenopus [30]–[32], [66] as well as chick [35], [67]. *Obelix* is weakly inducible by FGF, but its induction by a node cannot be blocked by SU5402, suggesting that the *Obelix*-inducing factor may be a different activator of the MAPK pathway such as IGF or PDGF. However, *TrkC* cannot be induced by FGF8 and its induction by a node is not abolished by SU5402, suggesting that FGF is neither necessary nor sufficient for *TrkC* induction. Wnt inhibition has been implicated as an additional signal, specifically acting to gate different responses to different levels of FGF [30], [66], [68]. However, the Wnt antagonist Dkk1 is also unable to induce expression of *Obelix*, as are retinoic acid, Somatostatin, Calcium, HGF/SF [69], [70], Cerberus [71]–[73] or BMP antagonists. Likewise, *TrkC* cannot be induced by retinoic acid, Calcium or Somatostatin. Since neither gene can be induced by any factor tested so far, it is likely that additional factors remain to be identified at least for the second phase (“streak group”) of the neural induction cascade. *Obelix* and *TrkC* therefore provide useful assays to identify this missing signal(s) (“X” and “Y” in Fig. 11).

Consistent with this conclusion, FGF is not sufficient to induce neural plate markers in non-prospective neural ectoderm, even in combination with BMP inhibitors [26], [27], [35], [74]. It is likely that even the unknown *Obelix*-/*TrkC*-inducing signals are insufficient, as suggested by analysis of the induction of *BERT*. Like *Obelix* and *TrkC, BERT* is not upregulated by FGFs, BMP antagonists, Wnt antagonists, Cerberus or HGF/SF [39]. However, BERT acts at a comparatively late point in the neural induction cascade, just before the onset of *Sox2* expression (green in Fig. 11), whereas *Obelix* and *TrkC* are both induced within the first 5 hours of this cascade. These findings suggest that at least two other signals, in addition to FGFs and BMP antagonism, may be involved in the acquisition of neural plate identity by epiblast cells – some of these are responsible for inducing *Obelix* and *TrkC* and acts as part of the “streak group” (second epoch) and another is responsible for inducing *BERT*, as part of the third epoch (“Z” in Fig. 11).

### Conclusions

The present study defines three genes as markers for early responses of epiblast cells to signals from Hensen's node, and their regulation. It strengthens the view that neural induction involves a cascade of sequential events, marked by genes deployed in a characteristic temporal sequence. Even the first 5 hours of signalling can be subdivided into two distinct steps: a “pre-streak” group of genes (induced within 3 hours and expressed before gastrulation in the normal embryo) a “streak group” (genes induced between 3–5 hours and expressed in the prospective neural plate after the mid-gastrula stage). The genes described here belong to the second group. One of them, *Asterix*, resembles all other previously studied genes of this group in that it is induced by FGF8, and that this factor is required for its induction by the organizer. FGF is neither necessary nor sufficient to induce *TrkC* and sufficient, but not necessary for induction of *Obelix*. These genes are also not induced by BMP antagonists, Wnt antagonists, Cerberus, HGF/SF, Calcium, retinoic acid or Somatostatin. These findings help us to define distinct steps in the neural induction cascade and provide new assays to identify the missing signals.

## Methods

### Isolation of full length clones for Asterix and Obelix

To isolate full-length cDNAs encoding the genes of interest, the initial partial clones obtained from the differential screen library [35], [36] were used to screen a stage 2–4 chick cDNA library (kind gift of J.C. Izpisua-Belmonte), directionally cloned in Uni-Zap (Stratagene) under standard stringent conditions (0.1 X SSC, 65°C). A radioactive probe was synthesized with Prime-IT II labelling kit (Stratagene) using 25–50 ng of the 550bp DNA fragment and 5 µl (50 µCi) [α-^32^P]dCTP. The unincorporated nucleotides were removed using a Probe Quant G-50 Micro Column (Pharmacia Biotech). The probe was then counted and 0.9×10^6^ cpm used per filter for library screening. 35×10^4^ clones were screened; positives were plaque-purified and converted into pBlueScript plasmids with ExAssistTM Interface-Resistence Helper Phage (Stratagene). Clones were then PCR amplified with T3-T7 primers and sequenced.

### Northern analysis

RNA was extracted from the area pellucida using Trizol Reagent (Invitrogen) following the manufacturer's protocol for small quantities of tissue, and separated by electrophoresis on formaldehyde gels, transferred by capillary elution to Nylon membranes (Amersham, Hybond-N+) as previously described [75]. The filter was then hybridized with denatured DIG-labelled RNA probe in DIG Easy Hyb (Roche; 100 ng/ml) for 16 hours at 68°C. After high-stringency washing, the probe was immunodetected with anti-digoxigenin-AP Fab' fragments (Roche) and revealed by chemiluminescence using CDP-Star (Roche).

### Subcellular localization in COS cells and in vivo

The subcellular localization of myc-tagged Obelix protein was determined in transfected COS cells and after electroporation in the epiblast of chick embryos. The open reading frame (ORF) of Obelix was cloned in pCDNA 3.1/Myc-His (Invitrogen), using the NotI and Hind III cloning sites. 10^5^ COS-1 cells were plated in a 35 mm cell culture dish and grown in DMEM containing 10% newborn calf serum. Transfection was performed using Lipofectamine Plus (Gibco BRL) following the manufacturers' instructions. After 48 hours' culture, cells were fixed with 4% formaldehyde and immunostained with anti-myc antibody (9E10) and either HRP-labelled goat anti-mouse IgG (Jackson), which was revealed with diaminobenzidine (DAB) or goat anti-mouse IgG-Cy3 (H + L) followed by fluorescence microscopy.

To study subcellular localization in vivo, Obelix-pCDNA3.1/Myc-His was cloned into the pCAβ vector (cut with BamHI and ClaI). This was used to electroporate embryos as described below, which were incubated 14-15 hours, fixed in 4% formaldehyde and stained as whole mounts with anti-myc antibody (9E10) as described above. The stained embryos were then embedded in paraffin wax and sectioned at 10 µm.

### Western blotting

10^5^ COS-1 cells were transfected with Obelix-pcDNA3.1/Myc-His as described above. The medium and cells were collected after 48 hours. Conditioned medium and cell extract were separated on 15% SDS-polyacrylamide gels under denaturing and reducing conditions, and proteins transferred onto a nitrocellulose membrane using a semi-dry blotter (Labconco) for 1 hour. Membranes were probed with anti-myc antibody (9E10) which was detected using goat-anti-mouse-IgG-HRP and LumiGLO (KPL).

### Embryos

Fertile hens' eggs (Brown Bovan Gold; Henry Stewart & Co. UK) and European quails' eggs were incubated at 38°C to the desired stage. Embryos were staged according to [76] (in Arabic numerals) or following Eyal-Giladi and Kochav for pre-streak stages (in Roman numerals) [42]. For experimental manipulations, embryos were cultured using a modified New culture method [77], [78]. Neural induction assays were performed as previously described [33], [79], [80]. For tissue grafts, quail donors and chick hosts were used. Factors were delivered either on carrier beads or by grafting transfected COS-1 cells.

FGFs were delivered using Heparin acrylic beads (Sigma) soaked for 2 hours in 5 µl of mouse recombinant FGF4 (0.1 µg/μl; R&D Systems), mouse recombinant FGF-8b (0.1 µg/μl; R&D Systems). Mouse recombinant HGF-SF (80 µg/μl; [69], [70] was delivered using AffiGel Blue beads. For SU5402, iononomycin and retinoic acid experiments, AG1X2 ion exchange beads (Formate form) were soaked in 25 µM SU5402 (CalBiochem), 2 µM ionomycin or 5 µg/ml all-trans retinoic acid (Sigma) (all in DMSO) for one hour at room temperature before grafting. For SU5402 experiments, three or four beads were grafted into the anterior area opaca and the embryos grown in New culture for 1 hour. A stage 3+ node was then grafted in close association with the beads and the embryos incubated for 6–16 hours.

Noggin was delivered using a stable Noggin-secreted cell-line [81] and the parent CHO cell line was used as a control for these cells (both gifts of Richard Harland), grafted as previously described [15]. Other factors were delivered using transfected COS cells. Cells were transfected using lipofectamine as described above, with the following constructs: HA-epitope tagged chordin in pMT21 [28], HA-tagged Cerberus in pcDNA3.1 [72], [73] and hDKK in pCS2++ [82].

Whole-mount in situ hybridization using digoxigenin (DIG)-labelled probes and immunocytochemistry were performed as previously described [83], [84]. When quail grafts were used, in situ hybridization was followed by immunostaining with QCPN antibody (maintained by the Department of Pharmacology and Molecular Sciences, The Johns Hopkins University School of Medicine, Baltimore, MD 21205 and the Department of Biological Sciences, University of Iowa, Iowa City 52242, under contract N01-HD-2-3144 from NICHD) using HRP and diaminobenzidine as described above.
